# Dietary l-carnitine Supplementation Modifies the Lipopolysaccharide-Induced Acute Phase Reaction in Dairy Cows

**DOI:** 10.3390/ani11010136

**Published:** 2021-01-10

**Authors:** Jennifer Meyer, Susanne Ursula Kononov, Sandra Grindler, Johanna Tröscher-Mußotter, Mohamad Taher Alaedin, Jana Frahm, Liane Hüther, Jeannette Kluess, Susanne Kersten, Dirk von Soosten, Ulrich Meyer, Erika Most, Klaus Eder, Helga Sauerwein, Jana Seifert, Korinna Huber, Anja Wegerich, Jürgen Rehage, Sven Dänicke

**Affiliations:** 1 Institute of Animal Nutrition, Friedrich-Loeffler-Institut, Federal Research Institute for Animal Health, Bundesallee 37, 38116 Braunschweig, Germany; Jennifer.Meyer@fli.de (J.M.); Susanne.Kononov@uni-hohenheim.de (S.U.K.); Liane.Huether@fli.de (L.H.); Jeannette.Kluess@fli.de (J.K.); Susanne.Kersten@fli.de (S.K.); Dirk.von_Soosten@fli.de (D.v.S.); Ulrich.Meyer@fli.de (U.M.); Sven.Daenicke@fli.de (S.D.); 2 Institute of Animal Science, Functional Anatomy of Livestock, University of Hohenheim, Fruwirthstraße 35, 70593 Stuttgart, Germany; S.Grindler@uni-hohenheim.de (S.G.); Korinna.Huber@uni-hohenheim.de (K.H.); 3 Institute of Animal Science, Functional Microbiology of Livestock, University of Hohenheim, Emil-Wolff-Str. 8, 70593 Stuttgart, Germany; Johanna.Troescher@uni-hohenheim.de (J.T.-M.); Seifert.Jana@uni-hohenheim.de (J.S.); 4 Institute for Animal Science, Physiology and Hygiene, Rheinische Friedrich-Wilhelms-Universität Bonn, Katzenburgweg 7-9, 53115 Bonn, Germany; Taherala@uni-bonn.de (M.T.A.); Sauerwein@uni-bonn.de (H.S.); 5 Institute of Animal Nutrition and Nutrition Physiology, Justus-Liebig-Universität Gießen, Heinrich-Buff-Ring 26-32, 35392 Gießen, Germany; Erika.Most@ernaehrung.uni-giessen.de (E.M.); Klaus.Eder@ernaehrung.uni-giessen.de (K.E.); 6 Clinic for Cattle, University of Veterinary Medicine Hannover, Foundation, Bischofsholer Damm 15, 30173 Hannover, Germany; anja.wegerich@tiho-hannover.de (A.W.); Juergen.Rehage@tiho-hannover.de (J.R.)

**Keywords:** l-carnitine, lipopolysaccharide, systemic inflammation, dairy cow, energy metabolism, clinical score, performance, non-esterified fatty acids, insulin

## Abstract

**Simple Summary:**

l-carnitine might limit the mitochondrial energy generation from fatty acids, particularly in periods of enhanced energy requirement such as during lipopolysaccharide (LPS) mediated innate immune responses. This study examined the influence of a dietary l-carnitine supplementation on dairy cows in mid-lactation which were challenged by an injection with LPS. Results suggest that this intervention supported the energy metabolism of the cows during LPS-induced acute phase reaction (APR).

**Abstract:**

l-carnitine plays an important role in energy metabolism through supporting the transport of activated fatty acids to the subcellular site of β-oxidation. An acute phase reaction (APR) is known as an energy consuming process. Lipopolysaccharides (LPS) are often used in animal models to study intervention measures during innate immune responses such as APR. Thus, the aim of the study was to investigate the effects of dietary l-carnitine supplementation during an LPS-induced APR in mid-lactating German Holstein cows. Animals were assigned to a control (CON, n = 26) or l-carnitine group (CAR, *n* = 27, 25 g rumen-protected l-carnitine/cow/d) and received an intravenous injection of LPS (0.5 μg/kg body weight) at day 111 post-partum. Blood samples were collected from day 1 pre-injection until day 14 post-injection (pi). From 0.5 h pi until 72 h pi blood samplings and clinical examinations were performed in short intervals. Clinical signs of the APR were not altered in group CAR except rumen motility which increased at a lower level compared to the CON group after a period of atonia. Group CAR maintained a higher insulin level compared to group CON even up to 72 h pi which might support glucose utilization following an APR.

## 1. Introduction

l-carnitine as a key factor in the carnitine palmitoyl-transferase system represents a precondition for the transport of fatty acids through the inner mitochondrial membrane into the matrix where the β-oxidation—one of the stages of energy generation from fatty acids through oxidative phosphorylation—takes place [1]. The dietary intake, endogenous synthesis and the reabsorption of l-carnitine including its precursors—N^Ƹ^–tri-methyl-lysine (TML) and y-butyro-betaine (yBB)—influence the l-carnitine status in mammals [2]. Different experiments indicated that an l-carnitine supplementation in the transition period of dairy cows supported the energy metabolism, more specifically the lipid metabolism as indicated by a decreased liver lipid accumulation and increased plasma triacyl-glyceride concentration (TG) [3,4]. Besides transition period, only limited information is available on the role of l-carnitine in other challenging, energy consuming situations in the life of dairy cows such as during an acute phase reaction (APR).

The APR is an innate immune response which can be triggered, amongst many other factors, by infections with gram-negative bacteria and an associated endotoxemia characterized by the presence of lipopolysaccharides (LPS) in the systemic blood circulation (sepsis) [5]. Based on this mechanism, an artificial systemic LPS administration is often used in animal models to study the effects of nutrients or intervention measures on the energy status and changes in blood parameters under the conditions of an APR.

Evidence was produced that many LPS-induced effects in dairy cows are related to the energy metabolism where l-carnitine is involved. A dose-dependent increase of plasma insulin concentration and a dose-dependent decrease of β-hydroxybutyrate (BHB) were detected in dairy cows challenged with four different LPS doses [6]. Additionally, it is known that an intravenous LPS injection is followed by an increase in plasma tumor necrosis factor-α (TNF-α) [6] as the main driver of the APR in dairy cows. Amongst others, TNF-α induces a rise in plasma concentration of non-esterified fatty acids (NEFA) and triacyl-glycerides (TG) [7]. Furthermore, a sufficient energy supply of immune cells is described as a precondition for their adequate function [8]. The energy supply hinges on an appropriate carnitine-dependent fatty acid shuttling and interestingly TNF-α affected the expression of l-carnitine transporters and the uptake of l-carnitine in bovine kidney cells [9]. This study was designed to determine the effects of a dietary l-carnitine supplementation on LPS challenged pluriparous dairy cows during mid-lactation. We hypothesized that l-carnitine supplemented dairy cows would benefit from a supported energy metabolism indicated through clinical-chemical and performance traits, and by a better mastery of the APR-associated clinical signs.

## 2. Materials and Methods

The experiment was carried out at the experimental station of the Institute of Animal Nutrition, Friedrich-Loeffler-Institut (FLI), in Braunschweig, Germany in accordance with the German Animal Welfare Act approved by the LAVES (Lower Saxony Office for Consumer Protection and Food Safety, Oldenburg, Germany) (AZ33.19-42502-04-16/2378).

### 2.1. Experimental Design

In this experiment 53 pluriparous German Holstein cows, including 7 rumen- and duodenum-cannulated animals, were assigned to a control (CON; *n* = 26, 3 of them cannulated) or an l-carnitine group (CAR; *n* = 27, 4 of them cannulated). The cows in CAR received 125 g of a rumen-protected l-carnitine product (Carneon 20 Rumin-Pro, Kaesler Nutrition GmbH, Cuxhaven, Germany) per cow and day, which was included in the feed concentrate. The daily l-carnitine intake was adjusted to 25 g per cow and per day. To compensate the fat content of the mentioned l-carnitine product, cows in CON received a rumen-protected fat product (BergaFat F-100 HP, Berg + Schmidt GmbH & Co. KG, Hamburg, Germany) of equal quality and quantity. Both groups were fed with 50% of a partial mixed ration (PMR) composed of 70% maize silage, 30% grass silage and 50% concentrate based on dry matter according to the recommendations for nutrient and energy supply of the Society of Nutrition Physiology (GfE, 2001). The PMR was offered in feed-weigh troughs (Roughage Intake Control (RIC) System Insentec B.V., Marknesse, The Netherlands) and each cow received the pelleted concentrate via electronic feeding stations (Insentec B.V., Marknesse, The Netherlands) individually restricted to their requirement. Water was offered ad libitum.

The supplementation of CON and CAR started at day 42 ante partum (ap). Groups of cows were balanced for numbers of lactation, body weight (BW), body condition score (BCS) and fat-corrected milk yield of previous lactation. The impact of the dietary l-carnitine supplementation during the transition period, covering the time between days 42 ap and day 110 post-partum (pp), was published earlier (Meyer et al. [4]).

**Figure 1:**
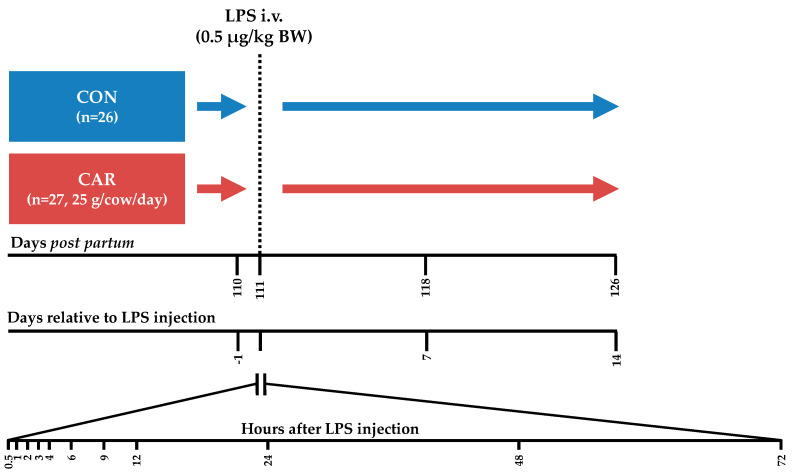
Experimental design. Cows in the control group (CON) as well as cows in the l-carnitine supplemented (CAR) group received an intravenous (i.v.) bolus injection of 0.5 μg/kg body weight (BW) lipopolysaccharides (LPS) on day 111 post-partum. Blood samples were collected until 14 days after LPS injection and frequently at the presented time points.

The present part of the experiment, which included the immune challenge, covered the period from day 110 pp until day 126 pp. Cows were housed in a free stall barn with slatted floors and stalls with rubber bedding. From day 110 pp until day 114 pp each cow was housed in a calving pen with straw bedding to supervise them closely during the LPS challenge. Each cow received an intravenous bolus injection of 0.5 μg/kg BW LPS (*E. coli*, Serotyp O111:B4, Sigma Aldrich, L2630, St. Louis, Missouri, USA) into the *Vena jugularis externa* without indwelling catheter on day 111 post-partum. In the following 72 h (h) post LPS injection (pi), samplings were performed frequently (Figure 1). To facilitate a high frequency of blood samplings around the LPS injection, cows were intravenously catheterized on day 1 ante injectionem (ai). During fixation in the feeding fence an indwelling catheter (2.4 mm × 200 mm Teflonkatheter, Walter Veterinär-Instrumente e.K., Baruth/Mark, Germany) was inserted in a *Vena jugularis externa* and closed with a stylet (Mandrin, Walter Veterinär-Instrumente e.K., Baruth/Mark, Germany). A few minutes ai the stylet was removed and replaced by a flex extension (Heidelberger-Verlängerung 75 cm, Tierärztebedarf J. Lehnecke GmbH, Schortens, Germany), which was equipped with a three-way valve (WDT Pharmazeutische Handelsgesellschaft mbH, Garbsen, Germany). After the blood sampling at 12 h pi the catheter was removed.

The components and chemical compositions of roughages and concentrates were previously published by Meyer et al. [4].

### 2.2. Measurements and Sample Collection

Samples of the PMR were taken daily, whereas the concentrate feed was sampled once a week. The feed samples were pooled over four periods. Cows were equipped with ear transponders to enable recording of the individual feed and water intake from weighing troughs and concentrate feeding stations. Feed and water were permanently offered for ad libitum consumption. During housing in the calving pens, water intake could not be recorded. 

A scale between milking parlor and stable recorded the BW twice a day after every milking time. The BCS was evaluated according to Edmonson et al. [10] once a week always by the same person. Daily milking was performed at 5:30 a.m. and 15:30 p.m. A milk counter (Lemmer Fullwood GmbH, Lohmar, Germany) recorded milk yield, and milk samples were taken twice a week during morning and evening milking to analyze milk ingredients.

The blood samples were obtained from *Vena jugularis externa* by needle puncture or by indwelling jugular catheters for the frequent sampling pi (from 0.5 h until 12 h pi). Prior to blood collection from the catheter, 20 mL blood was aspirated and discarded, and the catheter was flushed with 20 mL of 0.9% sterile saline solution after collection. Blood samples were taken at the following time points: day 110 pp (=day 1 ai), day 111 pp at 0.5, 1, 2, 3, 4, 6, 9, 12, 24, 48, 72 h pi; day 118 pp (=day 7 pi) and day 126 pp (=day 14 pi).

Clinical examinations of each cow were frequently performed from about 1 h ai until 72 h pi according to the methods of Dirksen et al. [11]. Gathered clinical findings were evaluated by a cumulative clinical score (modified from Meyer et al. [4]) by assigning the physiological level to 0 and deviations to increasing numbers (Table A1). Finally, the individual scores were cumulated and related to the maximum possible score of 31 and then converted to percentages. Thus, a score of 31 (=100%) represents a cow which deviated at maximum from the physiological value for each individual clinical parameter, while a score of 0 indicates a clinically inconspicuous cow. 

### 2.3. Analyses

The pooled feed samples were analyzed in accordance with the methods of the Association of German Agricultural Analytic and Research Institutes (VDLUFA) [12] for dry matter (method number 3.1), crude ash (method number 8.1), crude protein (method number 4.1.2), ether extract (method number 5.1.1), crude fiber (method number 6.1), neutral detergent fiber without ash (NDFom; method number 6.5.1) and acid detergent fiber without ash (ADFom; method number 6.5.2).

An infrared milk analyzer (Milkoscan FT 6000^®^; Foss Electric, Hillerød, Denmark) determined milk ingredient contents (fat, protein, lactose and urea), whereas a flow cytometric measurement (Fossomatic 500^®^, Hillerød, Denmark) determined the somatic cell count (SCC).

Total numbers of leukocytes were determined immediately after sampling in ethylene-diamine tetra-acetic acid (EDTA)-whole blood samples with an automatic analyzer (Celltac-α, MEK-6450, Nihon Kohden, Qinlab Diagnostik, Weichs, Germany). For NEFA, BHB, TG, cortisol and haptoglobin analyses serum was gained by centrifugation of blood (15 min, 15 °C, 1950× *g*, Varifuge 3.0, Heraeus, Hanau, Germany) after incubation for 30 min at room temperature and 30 min at 30 °C. Until the day of measurement, the extracted serum was frozen at a temperature of −80 °C. The Eurolyser CCA 180 (Eurolyser Diagnostica GmbH, Salzburg, Austria) performed the analyses of NEFA, BHB and TG by a photometric method. A sandwich-enzyme-linked immunosorbent assay (ELISA) was used to determine the insulin concentration (Bovine Insulin ELISA, 10-1201-01, Mecordia AB, Uppsala, Sweden) in serum according to the manufacturer’s protocol. Additionally, the serum haptoglobin concentration was determined using an ELISA method described previously [13] with a limit of detection of 0.07 mg/mL. Within the first 48 h pi, the concentration of serum cortisol was measured by radioimmunoassay (Cortisol RIA Kit, Beckman Coulter, Krefeld, Germany).

The determination of sodium, potassium, chloride, hydrogen carbonate and calcium ions, glucose and lactate concentration, temperature-corrected partial pressure of oxygen and carbon dioxide (TpO_2_, TpCO_2_), oxygen saturation (sO_2_), total carbon dioxide, temperature-corrected pH, base excess (BE) and base excess in extracellular fluid (Beecf) was performed by using an automated blood gas and electrolyte analyzer (GEM Premier 4000, Werfen, Kirchheim, Germany) in heparinized blood from sample syringes (Werfen, Kirchheim, Germany). A photometrical method (Calcium AS III Arsenazo III color test/Phosphorus (mono) ultraviolet (UV)-method kit, Greiner, Bahlingen, Germany) was used to analyze total calcium and total phosphorous in blood serum. A tandem mass spectrometry method according to Hirche et al. [14] was used to measure the concentration of TML, yBB, acetyl-carnitine (ACA) and free carnitine (CA) in EDTA plasma.

### 2.4. Calculations

Equations used for the calculation of fat-corrected milk (FCM), energy-corrected milk (ECM), milk energy, net energy requirement for maintenance (NEM), net energy requirement for lactation (NEL), net energy balance (NEB), feed efficiency (FE), Revised Quantitative Insulin Sensitivity Check Index (RQUICKI) and anion gap were previously published by Meyer et al. [4].

### 2.5. Statistical Analyses

Before statistical analyses weekly mean values were calculated for performance and feeding data. Additionally, daily mean values (_daily_) were calculated for some of the performance and feeding data from day 3 ai until day 3 pi. Analyses were performed using the MIXED procedure of SAS software (SAS Enterprise Guide 6.1, SAS Institute Inc., Cary, NC, USA) with the restricted maximum likelihood method (REML). As fixed factors the model included the group (G, CON or CAR), the time relative to LPS injection (T) and the interaction between G and T. The statistical analysis for the cumulative dry matter intake included the time points −1, 12, 24, 48 and 72 h pi. 

The choice of covariance structure (compound symmetry, autoregressive or unstructured) for the respective parameter was based on the smallest Akaike information criterion for a finite sample size (AICC). Effects were assumed as significant when *p*-Values were equal or smaller than 0.05. Least Square (LS) Mean comparisons were carried out by post hoc procedure (Tukey-Kramer). All results are given as LS-Means complemented by the standard error. In tables in “Appendix A” the pooled standard error (PSE) of LS-Means over both groups and all time points is indicated. 

## 3. Results

Initially, CON included 26 and CAR 27 cows. One animal of each group was excluded from the LPS challenge due to an initial rectal temperature lower than the lower bound reference temperature. Furthermore, one cannulated cow in CON died 24 h pi in cause of an acute shock related to undetected inflammation. Finally, 50 cows (n_CON_ = 24, n_CAR_ = 26) finished the trial.

As each cow served as its own control for evaluating the APR, the changes over time generally included the responses to LPS. Thus, the LPS effect is covered by the fixed factor of time. Results, which are not presented in this section, are presented in Appendix A.

### 3.1. Feed Intake, Body Condition and Milk

**Figure 2:**
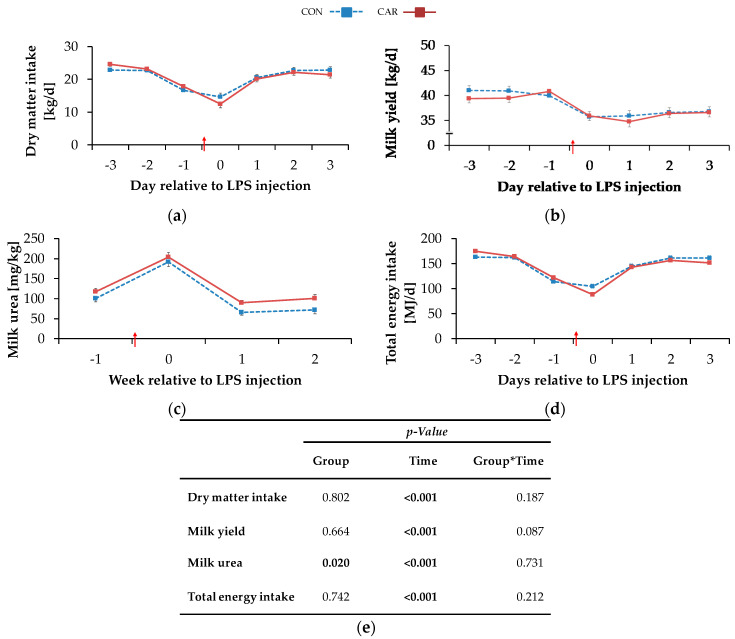
Progression of performance and feed intake data of dairy cows fed a non-supplemented (CON) or an l-carnitine supplemented diet (CAR, 25 g/d from 6 weeks ap up to day 126 pp) before and after an intravenous injection of 0.5 μg/kg BW lipopolysaccharides (LPS) on day 111 pp (red arrow). (**a**) Daily dry matter intake, (**b**) daily milk yield, (**c**) milk urea, (**d**) daily total energy intake, (**e**) data statistics. Data are given as LS-Means standard errors. Significant *p*-values are marked as bold.

The highest DMI_daily_ of 23.7 ± 0.4 kg/d occurred on day 3 ai and decreased by 43% to a minimum level of 13.6 ± 0.8 kg/d on the day of LPS injection. More specifically, all cows experienced a period of anorexia, which lasted for a mean time of 7 h and 49 min (1 h and 26 min–22 h and 23 min) in CON and for a mean time of 7 h and 55 min (00 h and 43 min–17 h and 19 min) in CAR after LPS injection (no significant difference). Until 12 h pi cows in CON reached 41% and cows in CAR 33% of the mean daily feed intake prior to LPS injection. Two days pi, DMI_daily_ returned completely to the initial level and was kept constant until day 3 pi (p_T_ < 0.001) (Figure 2a). Irrespective of treatments, water intake (Table A2) increased by 8% from week 1 ai to week 2 pi (no measurements in the week of LPS injection) and this level was maintained until the end of the experiment (p_T_ < 0.001).

The BW (Table A3) dropped independently of l-carnitine supplementation from week 1 ai until the week of LPS injection by 2% to a minimum level of 626 ± 9 kg. It then rose again to its initial level which remained unchanged until week 3 pi (p_T_ < 0.001). The BCS (Table A3) was not significantly influenced by treatments and amounted to 3.0 ± 0.1 on average. Irrespective of feeding group, milk yield (Table A3) reached a minimum level of 36.9 ± 0.6 kg/d in the week of LPS injection equivalent to a decrease by 9% (p_T_ < 0.001). Until the following week there was an increase, but milk yield did not return to the initial level until the end of the study. The daily resolution revealed an initial milk yield _daily_ (Figure 2b) of 40.4 ± 0.7 kg/d which was kept constant until day 1 ai. Thereafter, a decrease by 11% on the day of LPS injection was noticed and this level was kept until day 3 pi. Cows reached maximum milk fat values (Table A3) of 4.0 ± 0.10% in the week of LPS injection after an increase by 13% independently of the l-carnitine supplementation. Afterwards, milk fat returned to the initial level until week 2 pi and this level was kept until week 3 pi (p_T_ < 0.001). Also, for milk protein (Table A3), a time-dependent variation was detected (p_T_ <0.001) and resulted in a minimum value of 3.1 ± 0.0% in the week of LPS-injection. After a slight increase by 4% until week 2 pi, milk protein remained unchanged until the end of the trial. Therefore, fat-protein ratio (FPR) (p_T_ < 0.001) increased until the week of LPS injection to a maximum level of 1.28 ± 0.03 and returned to initial level until week 3 pi (Table A3). Milk lactose (Table A3) was neither affected by l-carnitine supplementation nor by the time relative to LPS injection. Milk urea (Figure 2c) was affected by l-carnitine supplementation (p_G_ = 0.020) and the average urea content of milk in CAR (128 ± 6 mg/kg) was 18% higher than in CON group (108 ± 6 mg/kg). Both groups reached their maximum level in the week of LPS injection and, after a decrease by 66% (CON) or 56% (CAR), their minimum level in week 2 pi. This level was kept in the following week. SCC (Table A3) seemed to be affected by the LPS injection (p_T_ = 0.054). Precisely, it increased by 9% from initial level until the week of LPS injection but returned to initial level already in week 1 pi. FCM (Table A4) decreased independently of feeding group from maximum level in week 1 ai by 7% to minimum level of 35.0 ± 0.7 kg/d in week 2 pi (p_T_ < 0.001) and returned to initial level until week 3 pi. ECM (Table A4) was unaffected by l-carnitine supplementation and differed in the same manner as FCM.

### 3.2. Energy Metabolism 

Total energy intake (Table A2) changed over time irrespective of l-carnitine supplementation and reached its minimum level of 152 ± 4 MJ NEL/d in the week of LPS injection (p_T_ = 0.003). Then it rose by 9% to the maximum level in week 3 pi. Daily resolution resulted in a total energy intake_daily_ (Figure 2d) of 168 ± 2 MJ NEL/d which dropped to 96 ± 6 MJ NEL/d at the day of LPS injection and increased by 63% until day 3 pi (p_T_ < 0.001). NEB (Table A2) reached its minimum level of −2.1 ± 3.9 MJ NEL/d in the week of LPS injection. In the following, NEB increased and reached at 2 weeks pi a level which was 10-fold higher than the initial level (p_T_ < 0.001). Also, NEL (Table A2) was unaffected by l-carnitine supplementation and decreased continuously by 6% from maximum level in week 1 ai to minimum level of 116 ± 2.10 MJ/d in week 2 pi (p_T_ = 0.002). Until week 3 pi, NEL returned to initial level. Milk energy (Table A4) increased from initial level by 5% to maximum level of 3.1 ± 0.1 MJ/kg in the week of LPS injection. Thereafter, it decreased and returned to initial level already 1-week pi (p_T_ < 0.001). Independently of the supplementation, feed efficiency (Table A2) decreased from 1-week ai until 2 weeks pi by 6% to the minimum value of 1.6 ± 0.0 kg/kg (p_T_ < 0.001).

### 3.3. Clinical Findings and Clinical Cumulative Score 

**Figure 3:**
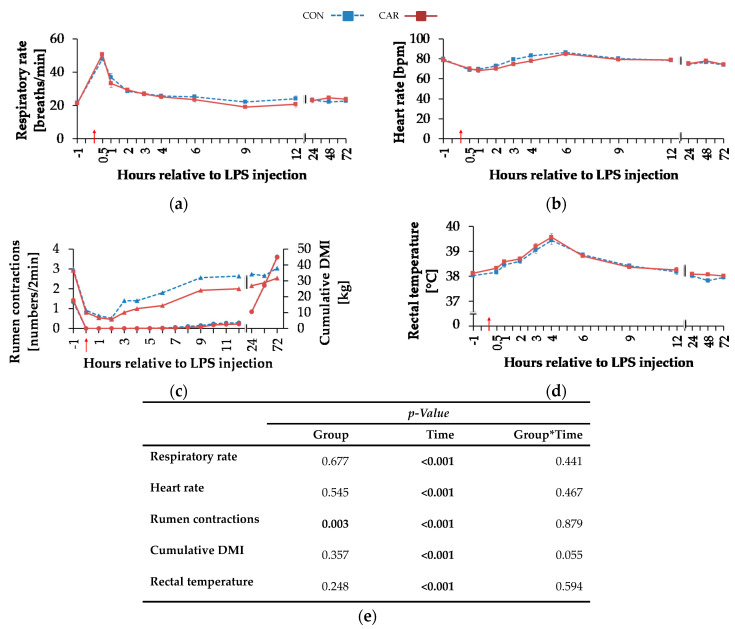
Progression of clinical parameters and cumulative dry matter intake of dairy cows fed a non-supplemented (CON) or an l-carnitine supplemented diet (CAR, 25 g/d from 6 weeks ap up to day 126 pp) before and after an intravenous injection of 0.5 μg/kg BW lipopolysaccharides (LPS) on day 111 pp (red arrow). (**a**) Respiratory rate (**b**) heart rate, (**c**) primary rumen contractions (triangles, left ordinate) and cumulative dry matter intake (DMI) (circles, right ordinate), (**d**) rectal temperature, (**e**) data statistics. Data are given as LS-Means standard errors. Significant *p*-values are marked as bold.

**Figure 4:**
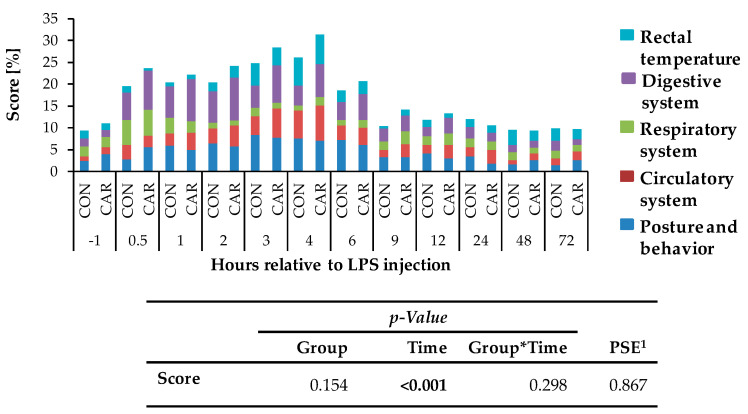
Cumulative clinical score (maximum reachable score: 31 points/100%), consisting of percentages of different categories (posture and behavior, circulatory, respiratory, digestive system and rectal temperature) of cows fed a non-supplemented (CON) or a l-carnitine supplemented diet (CAR, 25 g/d from 6 weeks ap up to day 126 pp) before and after an intravenous injection of 0.5 μg/kg BW lipopolysaccharides (LPS) on day 111 pp. Data are given as LS-Means. ^1^ Pooled standard error is listed. Significant *p*-values are marked as bold.

Independently of l-carnitine supplementation, initial respiratory rate (Figure 3a) rose by 130% and reached its maximum level of 49 ± 2 breaths/min 0.5 h pi which was followed by a 58% drop to minimum level until 9 h pi. This level remained unchanged until 72 h pi (p_T_ < 0.001). Heart rate (Figure 3b) decreased by 13% from initial level until 1 h pi where the minimum level of 69 ± 1 bpm was reached. Until 6 h pi the maximum of 86 ± 1 bpm occurred (significantly higher than the initial level) but decreased again by 14% until 72 h pi (p_T_ < 0.001). The numbers of primary rumen contractions (Figure 3c) were affected by the l-carnitine supplementation (*p*_G_ = 0.003) and the LPS injection (p_T_ < 0.001). In CAR, 1.6 ± 0.1 numbers/2 min were counted which was 20% lower than those in CON (1.9 ± 0.1 numbers/2 min). Interestingly, the primary rumen contractions in CAR reached the initial level already at 6 h pi, whereas in CON the return occurred at 9 h. Irrespective of treatment groups, the rectal temperature (Figure 3d) reached its maximum value of 39.5 ± 0.1 °C 4 h pi which corresponded to an increase by 4%. Following, it dropped by 4% until 72 h pi (p_T_ < 0.001). Independently of treatment groups, the cumulative clinical score (Figure 4) was twice as high at 0.5 h pi compared to ai due to a significant increase in all included scheme complexes except for rectal temperature. Especially the decreased numbers of rumen contractions and the incipient diarrhea resulted in an increase of the digestive complex. The score remained constant until 3 h pi and increased again until 4 h pi to the maximum value of 29 ± 1.4 due to an increase in the complex “rectal temperature”. The score dropped rapidly thereafter until 9 h pi back to the initial level which was kept constant until 72 h pi (p_T_ < 0.001).

### 3.4. Blood Parameters

#### 3.4.1. Carnitine

**Figure 5:**
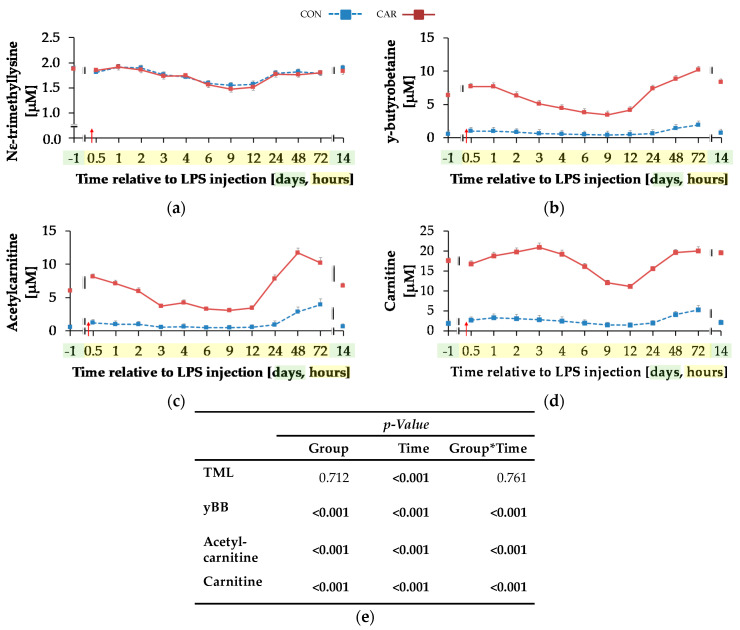
Progression of carnitine metabolites in plasma of dairy cows fed a non-supplemented (CON) or an l-carnitine supplemented diet (CAR, 25 g/d from 6 weeks ap up to day 126 pp) before and after an intravenous injection of 0.5 μg/kg BW lipopolysaccharides (LPS) on day 111 pp (red arrow). (**a**) Concentration of N^Ƹ^–tri-methyl-lysine (TML), (**b**) y-butyro-betaine (yBB), (**c**) acetyl-carnitine; (**d**) free carnitine. (**e**) Data statistics. Data are given as LS-Means standard errors. Significant *p*-values are marked as bold.

For TML (Figure 5a) a time-dependent variation was detected (p_T_ < 0.001). There was an initial decrease by 20% until 9 h pi where the minimum value of 1.52 ± 0.04 μM was reached, which was followed by an increase of 23% until day 14 pi. yBB (Figure 5b) was differently affected by treatment over time (p_G*T_ < 0.001). The initial level of yBB in CON (0.57 ± 0.54 μM) was kept constant until the end of the trial. In CAR, there was a decrease from the initial level until 9 h pi by 46% to the minimum level of 3.46 ± 0.54 μM. Thereafter, at 72 h pi the maximum level of 10.17 ± 0.53 μM was reached. In the following, yBB dropped again to initial level on day 14 pi. ACA (Figure 5c) was also differently affected by l-carnitine supplementation over time (p_G*T_ < 0.001). On average it was six times higher in CAR than in CON. The initial level in CON was 0.59 ± 0.34 μM and this level was restored by the end of the trial except for 72 h pi where the maximum level was reached. In CAR, ACA increased from day 1 ai until 0.5 h pi by 34% to a first peak. Thereafter, the minimum level of 3.09 ± 0.35 μM was reached at 9 h pi which was followed by an increase to a maximum level of 11.71 ± 0.69 μM until 48 h pi. Thereafter, ACA dropped by 42% until day 14 pi. Also, plasma concentration of CA (Figure 5d) was differently affected by l-carnitine supplementation over time (p_G*T_ < 0.001). Whereas initial level in CON (1.89 ± 0.83 μM) remained unchanged over the whole observation period, in CAR the CA concentration was seven times higher on average, dropped from day 1 ai until 12 h pi to a minimum value of 11.14 ± 0.57 μM and rose thereafter by 76% until 48 h pi. This level was kept until day 14 pi.

#### 3.4.2. Parameters Primarily Related to Energy Metabolism

**Figure 6:**
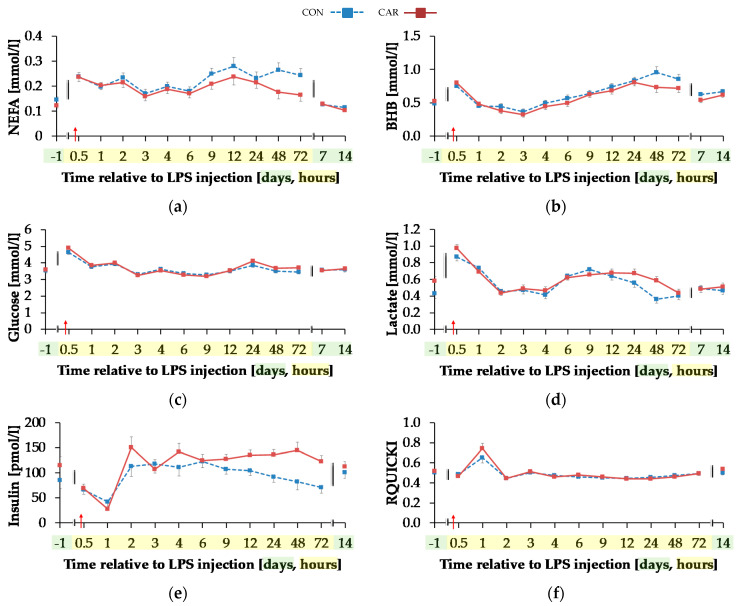

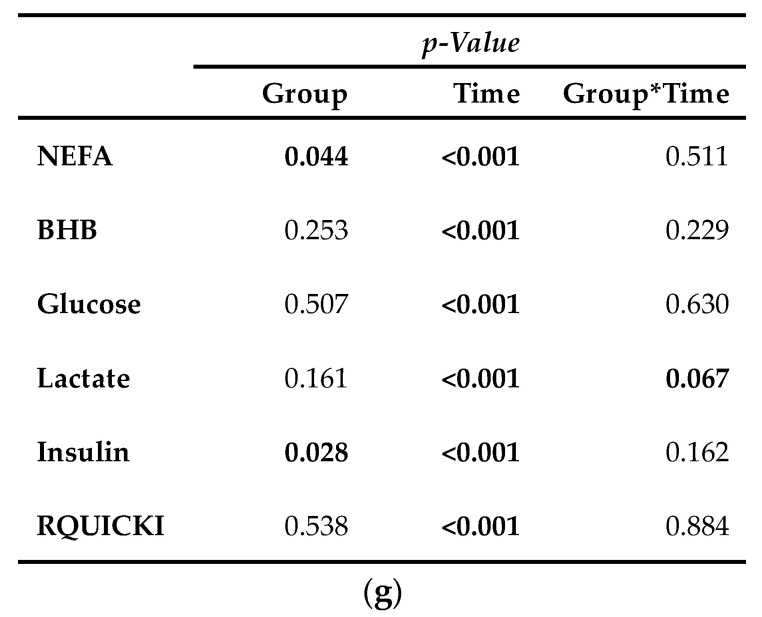
Progression of blood parameters with relevance for energy metabolism of dairy cows fed a non-supplemented (CON) or an l-carnitine supplemented diet (CAR, 25 g/d from 6 weeks ap up to day 126 pp) before and after an intravenous injection of 0.5 μg/kg BW lipopolysaccharides (LPS) on day 111 pp (red arrow). (**a**) Concentration of non-esterified fatty acids (NEFA) and (**b**) β-hydroxybutyrate (BHB) in serum, (**c**) glucose and (**d**) lactate in whole blood, (**e**) insulin in serum and (**f**) Revised Quantitative Insulin Sensitivity Check Index (RQUICKI) = $\frac{1}{\mathrm{logInsulin}\;\left[\frac{\text{µ}U}{\mathrm{ml}}\right]+\mathrm{logGlucose}\left[\frac{\mathrm{mg}}{\mathrm{dl}}\right]+\mathrm{logNEFA}\left[\frac{\mathrm{mmol}}{l}\right]}$ (**g**) Data statistics. Data are given as LS-Means standard errors. Significant *p*-Values are marked as bold.

Serum concentration of NEFA (Figure 6a) was on average 12% smaller in CAR than in CON (p_G_ = 0.044). The initial NEFA concentration increased by 63% in CON and by 92% in CAR until 0.5 h pi. In CON, a return to the initial level occurred at 1 h pi, whereas in CAR the initial NEFA concentration was reached again at 3 h pi. Irrespectively of the treatment groups, BHB (Figure 6b) rose by 53% from initial level to 0.5 h pi (p_T_ < 0.001). Until 3 h pi concentrations decreased to a minimum level of 0.35 ± 0.03 mmol/L and increased thereafter until 48 h pi to a maximum value which was 1.7-fold higher than the initial level. Until 7 d pi, BHB returned to the initial level. TG (Table A5) changed over time independently of l-carnitine supplementation (p_T_ < 0.001). There was an increase by 136% from day 1 ai until 0.5 h pi to the maximum value of 0.18 ± 0.01 mmol/L. Afterwards TG dropped until 1 h pi to a level, which was 30% smaller than the initial level. From there on, this smaller level was kept and returned to the initial level on day 7 pi.

Glucose concentration (Figure 6c) increased rapidly by 33% from day 1 ai until 0.5 h pi to the maximum level of 4.75 ± 0.08 mmol/L (p_T_ < 0.001). After a short decrease to the initial level, it rose again until 2 h pi to a second smaller peak followed by a decrease to the initial level which was kept constant until 12 h pi. Thereafter, an increase to another peak at 24 h pi was noticed which was equal to that at 2 h pi. Until the end of the trial, glucose concentration returned to the initial level. For lactate (Figure 6d) a time-dependent variation was detected (p_T_ < 0.001). There was a steep increase by 82% from day 1 ai until 0.5 h pi to maximum of 0.92 ± 0.03 mmol/L. Until 2 h pi lactate decreased to a value which was 12% lower than the initial level. It increased again until 9 h pi and at 24 h pi lactate concentration returned to initial level. Insulin levels (Figure 6e) were affected by l-carnitine supplementation and were on average 25% higher in CAR (116.33 ± 7.16 pmol/L) compared to CON (p_G_ = 0.028). The initial insulin concentration decreased by 65% until 1 h pi and returned to initial level at 2 h pi (p_T_ < 0.0001). This level was kept until the end of the trial. Independently of supplementation, RQUICKI (Figure 6f) reached the maximum value of 0.70 ± 0.03 after an increase by 36% 1 h pi. After a decrease to minimum level at 12 h pi which was 14% smaller than initial level, RQUICKI returned to initial level at 48 h pi (p_T_ < 0.001).

#### 3.4.3. Key Indicators for Acute Phase Reaction

**Figure 7:**
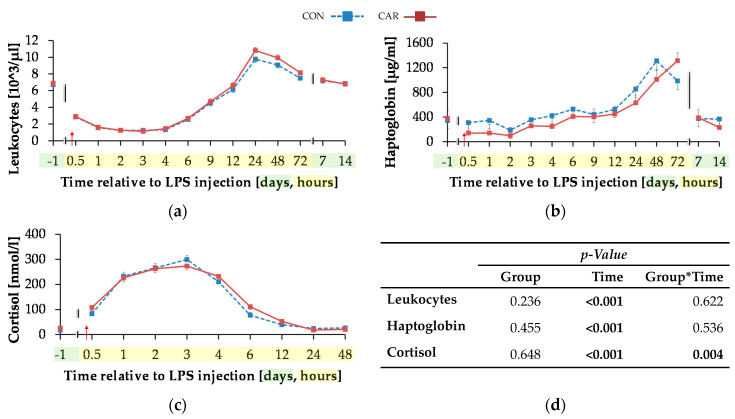
Progression of acute phase reaction related parameters of dairy cows fed a non-supplemented (CON) or an l-carnitine supplemented diet (CAR, 25 g/d from 6 weeks ap up to day 126 pp) before and after an intravenous injection of 0.5 μg/kg BW lipopolysaccharides (LPS) on day 111 pp (red arrow). (**a**) Total leukocyte count, concentration of (**b**) haptoglobin and (**c**) cortisol. (**d**) Data statistics. Data are given as LS-Means standard errors. Significant *p*-values are marked as bold.

The initial level of leukocytes (Figure 7a) was at 6.81 ± 0.19 × 10^3^/µL and the total numbers of leukocytes reached its minimum 3 h pi after a decrease of 83%. In the following, there was an increase to the maximum of 10.29 ± 0.28 × 10^3^/µL at 24 h pi (p_T_ < 0.001), which was 51% higher than the initial level. Up to 7 days pi the initial level was reached again and maintained until the end of the trial.

The initial serum haptoglobin concentration (Figure 7b) of 361 ± 92 µg/mL was kept up to 12 h pi. Afterwards, there was an increase to the maximum level of 1312 ± 161 µg/mL at 48 h pi, in CON group which was over three-fold higher than the initial level (p_T_ < 0.001). The CAR group reached the maximum of 1314 ± 128 µg/mL 72 h after LPS injection. At 7 d and d 14 pi the haptoglobin concentration complied the baseline level. 

The initial cortisol level (Figure 7c) of CON (17.88 ± 3.68 nmol/L) was statistically equal to that of CAR (25.08 ± 3.55 nmol/L). Both groups reached their maximum at 3 h pi (p_T_ < 0.001) and returned back to the initial level within the next nine h whereby the drop was more pronounced in the CON group (p_G*T_ = 0.004). Afterwards, the levels remained unchanged until the end of the trial.

#### 3.4.4. Blood Gases and Electrolytes 

**Figure 8:**
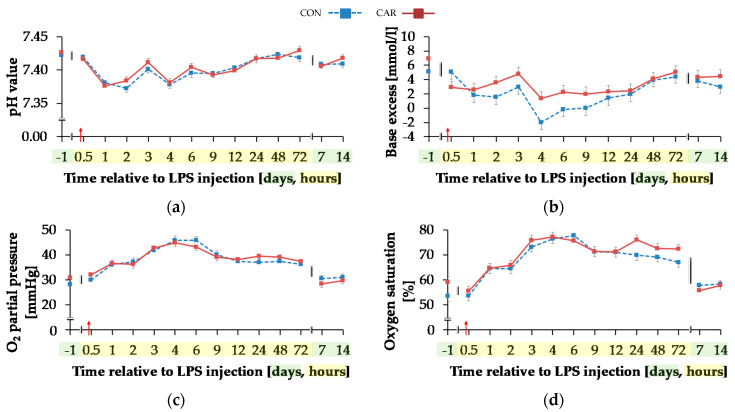

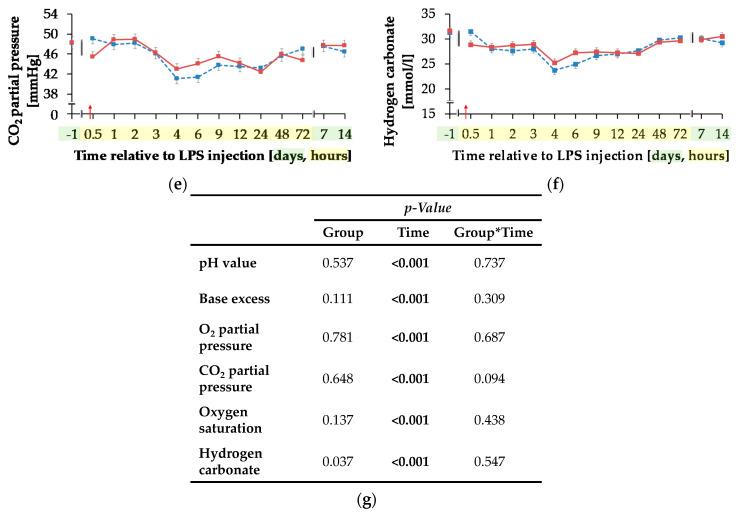
Progression of blood gas related parameters of dairy cows fed a non-supplemented (CON) or an l-carnitine supplemented diet (CAR, 25 g/d from 6 weeks ap up to day 126 pp) before and after an intravenous injection of 0.5 μg/kg BW lipopolysaccharides (LPS) on day 111 pp (red arrow). (**a**) pH value, (**b**) base excess, (**c**) O_2_ partial pressure and (**d**) oxygen saturation, (**e**) CO_2_ partial pressure and (**f**) Concentration of hydrogen carbonate in blood. (**g**) Data statistics. Data are given as LS-Means standard errors. Significant *p*-values are marked as bold.

Temperature corrected pH value (Figure 8a) decreased by 1% from initial and highest level to minimum level of 7.38 ± 0.004 until 2 h pi (p_T_ < 0.001) and 24 h pi the initial level was reached again. BE (Figure 8b) and Beecf (Table A5) behaved in the same manner (p_T_ < 0.001). Irrespective of treatment groups, they decreased by 63%/53% from initial and maximum level until 1 h pi. This level remained constant, but decreased from 3 h until 4 h pi to the lowest value of −0.32 ± 0.69 mmol/L/−0.04 ± 0.61 mmol/L. From 48 h pi on levels of BE and Beecf did not differ from the initial value.

Independently of treatment groups, TpO_2_ and sO_2_ (Figure 8c,d) increased by 54%/36% from initial to maximum level of 45.37 ± 1.05 mmHg/76.80 ± 1.34% 4 h pi (p_T_ < 0.001) and returned to initial level after 7 d pi. Also, for TpCO_2_ (Figure 8e) and total carbon dioxide (Table A5), a time-dependent variation was detected (p_T_ < 0.001). Lowest values occurred 4 h pi (42.05 ± 0.73 mmHg; 25.62 ± 0.58 mmol/L) and both parameters returned to the initial level 48 h pi. For hydrogen carbonate ions (Figure 8f) a time-dependent variation was detected (p_T_ < 0.001). From day 1 ai until 4 h pi minimum value of 24.48 ± 0.56 mmol/L was reached after a decrease by 22%. This increased level remained constant until 48 h pi when it returned to the initial level (p_T_ < 0.001) again.

**Figure 9:**
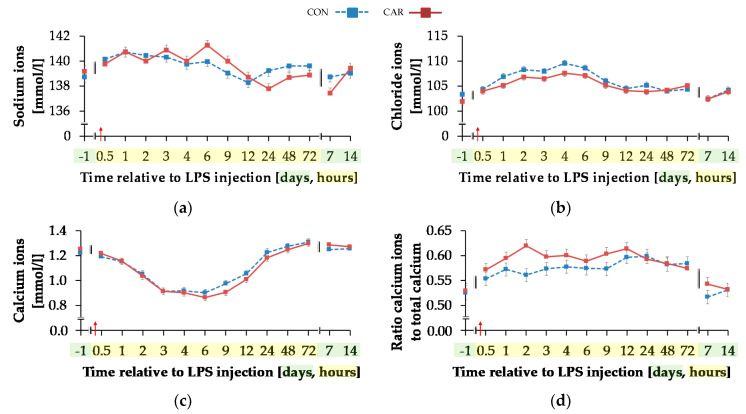

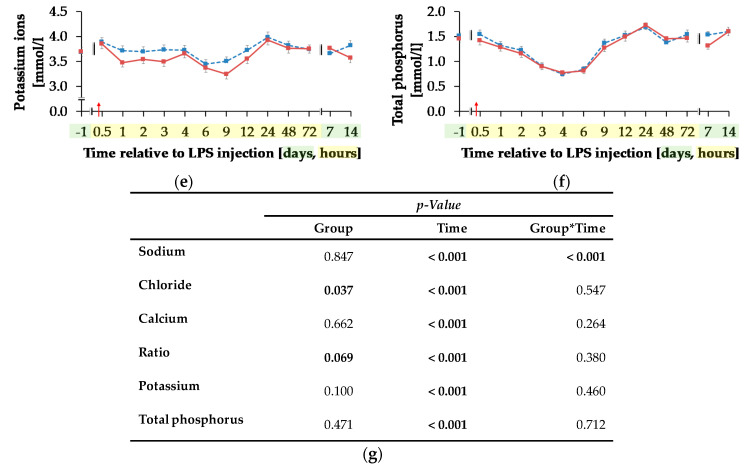
Progression of different electrolytes and total phosphorus in blood of dairy cows fed a non-supplemented (CON) or an l-carnitine supplemented diet (CAR, 25 g/d from 6 weeks ap up to day 126 pp) before and after an intravenous injection of 0.5 μg/kg BW lipopolysaccharides (LPS) on day 111 pp (red arrow). (**a**) Concentration of sodium ions and (**b**) chloride ions, (**c**) calcium ions and (**d**) the ratio of calcium ions to total calcium, (**e**) potassium ions and (**f**) total phosphorus in whole blood. (**g**) Data statistics. Data are given as LS-Means standard errors Significant *p*-values are marked as bold.

Blood sodium ion concentration (Figure 9a) was differently influenced by l-carnitine supplementation over time (p_G*T_ < 0.001). In CON, the initial level of 138.74 ± 0.46 mmol/L remained unchanged over the whole experiment. In CAR, sodium ions rose by 2% from initial to maximum level of 141.27 ± 0.40 mmol/L at 6 h pi. Thereafter, sodium ion concentration returned to initial level on day 14 pi. Chloride ions (Figure 9b) were 1% smaller in CAR compared to CON on average (p_G_ = 0.037). They reached a maximum level at 4 h pi after an increase by 6% from initial level and returned to it at 12 h pi (p_T_ < 0.001). Irrespective of treatment groups, calcium ions (Figure 9c) dropped by 29% from day 1 ai until 6 h pi to minimum level of 0.88 ± 0.02 mmol/L and returned to initial level until 48 h pi. The total calcium concentration (Table A5) also reached a nadir at 6 h pi but approached the initial level already 24 h pi (p_T_ < 0.001). The ratio of calcium ions to total calcium (Figure 9d) also varied with time irrespective of treatment groups. From day 1 ai until 2 h pi, an increase by 12% was detected. This increase level was kept and a second peak at 12 h pi represented the maximum value (p_T_ < 0.001). A return to initial level did not occur until day 7 pi. Total phosphorus (Figure 9f) declined by 48% from 0.5 h pi until 4 h pi to the lowest level of 0.76 ± 0.05 mmol/L and returned to initial level 9 h pi (p_T_ < 0.001). Independently of l-carnitine supplementation, ratio of total calcium to total phosphorous (Table A5) remained unchanged from day 1 ai until 3 h pi. Then it increased to its maximum level at 4 h pi, which was 48% higher than the initial level. After a decrease, the lowest value was reached 12 h pi and 48 h pi it returned to initial level. Potassium ions (Figure 9e) varied independently of l-carnitine supplementation and decreased by 9% from initial level until 9 h pi to the minimum value of 3.37 ± 0.06 mmol/L (p_T_ < 0.001). At 12 h pi the initial level was reached again (p_T_ < 0.001). Anion gap (Table A5) differed only slightly over time (p_T_ < 0.001). A minimum level of 8.11 ± 0.45 mmol/L was detected 2 h pi but there were no significant deviations from the initial level.

## 4. Discussion

The present experiment was designed to test the hypothesis that an l-carnitine supplementation improves the energy status of mid-lactating dairy cows during a period of a systemic inflammation induced by LPS with consequences for the clinical outcome and the time-dependent resolution of the APR.

An LPS challenge is known not only to induce an APR but also to downregulate l-carnitine transporter expression in mammary glands of lactating rats [15]. Furthermore, it is reported that an l-carnitine supplementation enhanced the acute phase protein response in LPS challenged broiler chickens [16] and that reduced carnitine concentrations in serum were related to immunological disorders in humans [17]. 

Anorexia, hyperthermia, dyspnea, dyscardia, leukopenia and leukocytosis, stress response (elevation of blood cortisol), synthesis of acute phase proteins (APP) like haptoglobin, ruminal hypomotility and atonia are cardinal symptoms of an APR in the bovine (e.g., Jacobsen et al. [18], Burdick et al. [19]) which were also observed in the present experiment, particularly during the first hours after LPS injection. The clinical manifestation of the APR is mediated by the release of pro-inflammatory cytokines such as TNF-α and interleukin 6 (IL-6) [20]. Besides triggering sickness behavior, the APR is associated with marked metabolic alterations, most notably with a metabolic acidosis as indicated by a decrease of blood pH and BE. In the present trial, the drop in pH was paralleled by a period of tachypnea aimed at respiratory compensation, which in turn enforced the decrease of TpCO_2_ through an increase of ventilation which also stressed the bicarbonate buffer system. The detected acidosis results, among other factors, from the *lactatemia* born from anaerobic metabolic pathways. These were partly triggered by hemodynamic effects resulting in an impaired oxygen tissue delivery due to mal-perfusion and ischemia of certain tissues and body regions. Thus, the steady increase in venous TpO_2_ might reflect a mismatch between tissue oxygen delivery and usage as demonstrated for septicemic dogs [21], possibly intensified by the tachypnea. However, the extent of this effect cannot be evaluated since only arterial TpO_2_ provides information on tissue oxygenation but not the venous TpO_2_. As arterial and venous TpO_2_ do not correlate with each other in septicemic human patients [22], pigs [23] and sheep [24] the simultaneous determination of both partial oxygen pressures would allow the calculation of the arterio-venous difference for a better evaluation of the peripheral oxygen consumption. While results of the blood gas analysis and indicators of the metabolic acidosis were not modified by l-carnitine supplementation, the LPS associated alterations in the electrolyte-water balance were subject to l-carnitine effects.

Besides the stagnating DMI and consequently calcium intake, the severe hypocalcemia (total calcium < 2 mmol/L) observed in the period between 2 and 12 h pi might be due to a redistribution of body calcium between blood and other body compartments, including possible APR-induced edema as described for pigs. On the other hand, the proportion of biologically active Ca^2+^ ions of total calcium increased at the same time although Ca^2+^ ions itself also decreased. This effect might be related to the decrease in blood pH, a situation when Ca^2+^ ions are displaced from albumin by H^+^ ions. This effect appeared to be more pronounced in CAR compared to CON although pH values displayed no group differences. At the same time, a time-dependent hyperchloremia was observed, which was less pronounced in CAR. Compared to all other electrolytes present in blood, chloride is the most abundant anion and is therefore regarded as a main regulator in acid-base balance which might be particularly important under septicemic conditions [25]. A hyperchloremia in endo-toxemic dogs suffering from metabolic acidosis with a parallel normo-natremia was attributed to differential shifts of Na^+^ and Cl^−^ ions from extravascular to vascular spaces [26]. The changes in Na^+^ were also less pronounced in the present experiment when compared to Cl^−^. A displacement of chloride by other anions such as bicarbonate which might also have got lost due to the observed diarrhea—besides its diminishment in the course of the respiratory compensation of the metabolic acidosis—might also induce hyperchloremia. Chloride levels started to decrease in CAR from 1 h pi onwards when also bicarbonate levels were higher compared to CON, which supports the idea of the anion exchange effects. As water intake clearly influences the body fluid distribution the reduced water intake following LPS challenge can be considered as an additional factor influencing concentrations. Nevertheless, the less pronounced hyperchloremia in CAR along with the slightly less pronounced BE and higher proportion of ionized calcium to total calcium suggest metabolic effects due to the l-carnitine supplementation. l-carnitine not only plays an important role in shuttling of fatty acids between the cytosolic and mitochondrial compartments but is also considered as an acetyl-buffer [2]. This might be particularly relevant under anaerobic conditions seen in septicemic conditions where the lack of oxygen results in an accumulation of acetyl-CoA which cannot be utilized by the tri-carboxic acid cycle [27]. Removal of acetyl-CoA prevents blocking of pyruvate dehydrogenase and thus lactate formation and furthermore releases CoA usable for further catabolic reactions. However, although ACA blood levels increased upon LPS challenge more pronouncedly in CAR compared to CON, the initial lactate peak was comparable between both groups suggesting that anaerobic glycolysis was not prevented. However, it needs to be stressed that only the extracellula*r* levels of l-carnitine and its metabolites in blood were recorded. Hence, the situation in tissues, particularly in muscle, could have been different. These putative metabolic effects of l-carnitine could have altered the intracellular acid-base milieu relative to the extracellular space with consequences for the electrolytes measured in blood. 

TNF-α mediated anorexia and decreased DMI are important features of the APR since nutrient and energy intake are reduced simultaneously with consequences not only for electrolyte balance but also for the entire metabolism. Neither DMI_daily_ nor total energy intake_daily_ were affected by the l-carnitine supplementation. The minimum of DMI_daily_ and total energy intake_daily_ confirmed the results of Waldron et al. [6], which indicated a transient decrease on the day of intravenous LPS infusion (*E. coli*, O111:B4, 0.5 μg/kg BW, over 100 min) in dairy cows in mid-lactation. Interestingly, DMI_daily_ as well as total energy intake_daily_ decreased already from day 1 ai, which might also be due to the rehousing of the cows. 

The role of TNF-α in mediating anorexia and gastric stasis was proposed to result from action within the neural circuitry of the medullary dorsal vagal complex (DVC) as experimentally shown in endo-toxemic rats [28]. Thus, the rumen hypomotility and temporal atonia as observed in the present experiment might have been directly triggered by TNF-α effects on the autonomic nervous system. Also, the temporary decrease of Ca^2+^ concentration in blood might be related to the observed variations in rumen contractions. The prolonged period of decreased primary rumen contractions in CAR might be related to the sustained insulin response in this group. Ali et al. [29] described an inhibition of primary rumen contractions during non-ruminating and non-eating periods after an intravenous injection of 1 IU insulin/kg BW in mature Clun Forest sheep. They hypothesized that this inhibition resulted from the emerging hypoglycemia because of the disappearance of this effect after glucose injection. In contrast to the study of Ali et al. [29], the present study showed no period of hypoglycemia in the mid-lactating cows. Interestingly, ACA is known for its influence on the peripheral nervous system in humans [30] and according to Fregonesi et al. [31] an ACA supplementation of diabetic rats was related to a diminishment of areas of neuronal profiles in the stomach, which might indicate a neuroprotective effect. Therefore, an impact of l-carnitine supplementation on primary rumen contractions via the enteric nervous system also cannot be ruled out.

Among other things, the decrease in milk yield due to LPS challenge in the present study might not only be due to the reduction of energy intake (total energy intake_daily_). While milk yield remained at this lower level until the end of the trial (week 3 pi), the total energy intake returned to initial level already in week 2 pi suggesting a compromised energy utilization in post-endo-toxemic cows. This overall reduced energy utilization could hint at a prioritization of energy for resolution of APR instead of for milk energy excretion. In a study of Shuster et al. [32] the suppression of milk yield occurred also in untreated quarters and in the absence of a decrease of DMI in dairy cows in mid and late-lactation challenged by a mastitis induced by an infusion of 10 μg/kg LPS (*E. coli*, O55:B5) in two homolateral quarters of the udder. They concluded that the suppression of milk yield in all quarters during mastitis resulted from a systemic effect of inflammation. Thus, the reduced milk yield as observed in the present experiment might also be related to systemic inflammatory effects apart from the suppression of feed intake.

In contrast to the period around calving [4], milk urea was significantly higher in CAR than in CON in the present period of trial, despite a comparable level in the week before LPS injection. Roseler et al. [33] described a negative correlation between energy intake and plasma urea for healthy, multiparous Holstein cows in mid-lactation. In the present study the milk urea content also peaked due to LPS injection which coincided with the period of the most pronounced suppression of total energy intake. Accordingly, feed restriction in mid-lactating Holstein cows caused an increase in milk urea while the abomasal l-carnitine infusion (20 g/d/cow) had no impact under these feeding conditions [34]. Blood and consequently milk urea levels are also influenced by body protein turnover of the cow. An APR is known particularly to trigger muscle protein breakdown to meet the amino acid requirements for the hepatic synthesis of acute phase proteins, which is considered as a nitrogen wasting process due to amino acid patterns differing between acute phase proteins and muscle protein. As a result, unused amino acids are catabolized to urea which is also eliminated via milk. The sepsis-associated nitrogen wasting has been discussed as being connected with an endogenous l-carnitine depletion [35], and its dietary supplementation was reviewed to improve N-balance in humans and animals [36]. A temporal reduction of l-carnitine and its metabolites was also observed in the present experiment in the course of the endotoxemia. Interestingly, this drop occurred at a higher level and was more pronounced in the group supplemented with l-carnitine which underpinned its role in protein turnover. However, under the conditions of the APR in the present study, no indications for l-carnitine associated shifts in milk urea kinetics or an earlier return to the initial performance were detected.

In the study of Giri et al. [37] an intravenous LPS infusion of 2.5 μg/kg BW over 6 h in 10 pregnant cows at different stages of gestation resulted in an initial hyperglycemia from 1 h until 3 h of infusion and significant elevated plasma cortisol level from up to 2 h pi. Thereafter, a development of a hypoglycemia was detected. In the present study, there was an also increase from initial blood glucose concentration until 0.5 h pi, which might be due to alterations in the hepatic glucose metabolism (gluconeogenesis and glycogenolysis) which in turn might be a consequence of an elevated cortisol level due to the LPS challenge [38]. In contrast to the study of Giri et al. [37] no stage of hypoglycemia was observed in the present study, despite a reduced total energy intake during a period of an energy-consuming immune activation. According to Filkins et al. [39] the period of hypoglycemia resulted from a depression of the hepatic gluconeogenesis. Thus, the lack of a hypoglycemic period in the present trial might indicate a still sufficient level of gluconeogenesis/glycogenolysis.

The initial hyperglycemia was indicated by similar time-resolved patterns of serum NEFA and BHB contents. According to Kushibiki et al. [7], an intravenous injection of recombinant bovine TNF-α (5.0 μg/kg), mimicking an APR, resulted in an increase of NEFA concentrations in blood with a simultaneously unaffected DMI in Holstein heifers. The higher NEFA concentration in CON in comparison to CAR (*p* < 0.05) in the present experiment at similar BHB- and TG concentrations and performance data of both groups might hint at an increase of efficiency of β-oxidation in CAR. Carlson et al. [34] reported that neither an abomasal l-carnitine infusion of 20 g per cow per day in feed restricted or non-feed restricted mid-lactating Holstein nor a dietary l-carnitine supplementation of 6, 50 or 100 g per cow per day in transition period [40] affected plasma NEFA concentration. According to Buttgereit et al. [8], the function of immune cells in periods of negative energy balances is limited by their energy supply. Famularo et al. [17] also suggested that this energy supply depends, among other factors, on the availability of l-carnitine to oxidize fatty acids. Whether the lower NEFA levels in CAR indicate an increased oxidation of NEFA cannot be answered conclusively based on the recorded parameters.

The varying effects of l-carnitine supplementation on NEFA and insulin might be related to the energetic basic situation reflected by the extent of energy deficit. According to Mason et al. [41] an increase in plasma NEFA concentrations, as noticed after LPS injection in the present study, can decrease the glucose-reducing effect of insulin in rats. This effect was also detected in the present study. The significant increase of insulin concentration from 1 h until 2 h pi did not affect the glucose concentration during this period. Oikawa et al. [42] reported that this effect resulted from either a decreased insulin sensitivity and/or a reduced insulin responsiveness.

Cortisol is regarded as an early effector molecule in endotoxin-challenged cattle acting on the subsequent activity of the innate immune system, including the TNF-alpha response [43]. On the other hand, cortisol is known for its anti-inflammatory effects under the conditions of an APR [44]. According to Gross et al. [45] changes in blood concentrations of glucose and BHB are closely involved in the regulation of cortisol secretion, which resulted in changes of the pulsatile release of cortisol in intramammary LPS-challenged, mid-lactating cows. In the present study the initial increase of cortisol went along with alterations of glucose, insulin, NEFA, BHB and TG concentrations. Also, in the study of Waldron et al. [6] the cortisol concentration increased after an intravenous LPS injection in mid-lactating dairy cows paralleled by simultaneously increased glucose levels, depressed feed intake and increased circulating blood NEFA concentration. The decrease of BHB concentration in the present study (3 h pi) was also detected in a study of Waldron et al. [6]. They reported that plasma BHB decreased linearly after an injection of 0.5, 1.0 and 1.5 μg/kg BW LPS in dairy cows in mid-lactation and discussed that the BHB decrease was not clearly related to a decreased ketogenic metabolism of cells in the ruminal epithelium or a limited precursor supply caused by a drop in DMI after LPS injection. Werling et al. [46] and Steiger et al. [47] also reported a decreased BHB concentration in blood of dairy cows after an intravenous LPS injection of 2 μg/kg BW over a period of 100 min. According to Waldron et al. [6] et al. tissues might use BHB as alternate energy source due to the increase of glucose usage of immune cells during an LPS challenge.

The initial LPS-related and CAR-independent increase of TG in the present experiment is in accordance with the results of Kushibiki et al. [7]. They reported a hypertriglyceridemia in Holstein heifers after an injection of recombinant bovine TNF-α which was accompanied by a temporarily rise of very-low-density lipoprotein concentration and therefore a higher transport capacity from liver into blood circulation and peripheral tissues. 

## 5. Conclusions

A dietary l-carnitine supplementation of 25 g per day and cow did not alter the time course of clinical signs of an APR induced by an intravenous LPS-administration, except for rumen motility which increased at a lower level compared to the CON after a period of atonia. Whether the endotoxemia sensitized the enteric nervous system for putative inhibitory effects of acetyl-carnitine remains to be examined further.

The temporal resolution of the APR-associated metabolic acidosis was not modified by l-carnitine supplementation, while hyperchloremia was less pronounced in CAR hinting at a specific role of carnitine in electrolyte and water balance.

The most striking effect of l-carnitine supplementation was the higher insulin concentration in CAR compared to CON, which persisted even until 72 h after initiation of the APR. This relative hyperinsulinemia was concurred with lower NEFA levels and an unchanged insulin sensitivity suggesting an anti-lipolytic effect.

## Data Availability

All data presented in this study are available on request from the corresponding author.

## Appendix A

**Table A1:** Definition of cumulative clinical score. The physiological level has a score of 0. Clinical findings were assigned to five different categories. The lines below parameters indicate that only the parameter above or below adds to the cumulative score. A maximum of 31 points could be reached.

Category	Parameter	Score	Meaning
*Posture and behavior*	Ear position	0, 1, 2	Horizontal, tense and backwards, low
	Behavior		
	Calm	0, 1, 2	Calm and attentive, calm, apathetic
	Nervous	0, 1, 2	Calm and attentive, nervous, hyper nervous
*Circulatory*	Muzzle	0, 1	Wet, dry
	Conjunctive		
	Hypoaemic	0, 1, 2	Pale rose, pale, cyanotic
	Hyperaemic	0, 1, 2	Pale rose, rose red, red
	Injected episcleral vessels	0, 1, 2, 3	No, low, medium, severe injected
	Washed out episcleral vessels	0, 1	No, yes
	Heart rate		
	Bradycardia	0, 1, 2, 3	65–85, 44–64, 23–43, <43 [bpm]
	Tachycardia	0, 1, 2, 3	65–85, 86–106, 107–127, >127 [bpm]
*Respiratory*	Respiratory rate		
	Bradypnea	0, 1, 2, 3	24–36, 15–23, 6–14, <6 [breaths/min]
	Tachypnea	0, 1, 2, 3	24–36, 37–49, 50–62, >62 [breaths/min]
	Pathological breathing sound	0, 1, 2, 3	None, low, medium, severe labored breathing
*Digestive*	Primary rumen contractions		
	Hypoperistaltic	0, 1, 2	2–3, 1, 0 [numbers/2 min]
	Hyperperistaltic	0, 1, 2	2–3, 4, >4 [numbers/2 min]
	Rumen sound		
	reduced	0, 1	Loud, less loud-silence
	increased	0, 1	Loud, intensive loud
	Abdominal wall tension	0, 1, 2, 3	No, low, medium, severe increased tension
	Dorsal gas bubble	0, 1	Not existing, existing
	Diarrhoea	0, 1	No, yes
*Rectal temperature*	Rectal temperature		
	Hypothermia	0, 1, 2, 3	38–39, 37–37.9, 36–36.9, <36 [°C]
	Hyperthermia	0, 1, 2, 3	38–39, 39.1–40, 40.1–41, 41.1–42 [°C]

**Table A2:** Effects of an l-carnitine supplementation on water intake, total energy intake, net energy requirement for lactation (NEL), net energy balance (NEB) and feed efficiency of German Holstein cows challenged by an *intravenous* injection of lipopolysaccharides (LPS, 0.5 μg/kg BW) at the first day in week 1.

Week Relative to LPS Injection	Water Intake [kg/d]	Total Energy Intake [MJ NEL/d]	NEL ^1^ [MJ/d]	NEB ^2^ [MJ NEL/d]	Feed Efficiency
**CON/CAR**					
**−1**	75/71	159/161	124/123	−2.6/1.6	1.7/1.7
1		155/150	120/116	−2.2/−2.1	1.7/1.7
2	82/77	156/166	118/114	0.2/14.5	1.7/1.5
3	82/79	162/169	121/117	3.9/14.7	1.6/1.5
*p*-Value ^3^					
G	0.137	0.426	0.328	0.066	0.050
T	**<0.001**	**0.003**	**0.002**	**0.001**	**0.001**
G*T	0.663	0.192	0.847	0.107	0.144
**PSE** ^4^	1.825	2.902	2.210	2.931	0.034

Data are given as LS-Means for control group (CON) and l-carnitine group (CAR). No recording of water intake in week 1 (-), ^1^ NEL = (milk energy [MJ/kg] + 0.086) × milk yield [kg/d], ^2^ NEB = Total energy intake [MJ NEL/d] − (NEM [MJ NEL/d] + NEL [MJ NEL/d]), ^3^ *p*-Value for group (G), time (T) and their interaction (G*T), ^4^ pooled standard error. Significant *p*-values are marked as bold.

**Table A3:** Effects of an l-carnitine supplementation on body condition and milk parameters of German Holstein cows challenged by an *intravenous* injection of lipopolysaccharides (LPS, 0.5 μg/kg BW) at the first day in week 1.

WeekRelative to LPS Injection	Body Weight [kg]	BCS ^1^	Milk Yield [kg/d]	Milk Fat [%]	Milk Protein [%]	Fat-Protein Ratio	Lactose [%]	SCC ^2^ [log10/mL]
**CON/CAR**								
**−1**	647/636	3.09/2.99	41.3/39.6	3.4/3.6	3.2/3.2	1.08/1.14	4.83/4.88	1.40/1.57
1	629/623	3.05/2.92	37.2/36.5	4.1/3.9	3.2/3.1	1.29/1.26	4.91/4.75	1.61/1.62
2	645/635	3.01/2.92	40.2/38.2	3.3/3.3	3.2/3.3	1.00/1.02	4.88/4.86	1.58/1.57
3	648/638	3.05/2.95	40.5/38.4	3.3/3.6	3.3/3.3	1.01/1.10	4.87/4.86	1.52/1.57
*p*-Value ^3^								
G	0.610	0.424	0.172	0.591	0.819	0.502	0.549	0.709
T	**<0.001**	0.182	**<0.001**	**<0.001**	**0.001**	**<0.001**	**0.883**	**0.054**
G*T	0.373	0.862	0.109	0.132	0.564	0.270	0.298	0.265
**PSE** ^4^	9.311	0.067	0.634	0.112	0.040	0.034	0.044	0.079

Data are given as LS-Means for control group (CON) and l-carnitine group (CAR). ^1^ Body Condition Score, ^2^ Somatic Cell Count, ^3^ *p*-Value for group (G), time (T) and their interaction (G*T), ^4^ pooled standard error. Significant *p*-values are marked as bold.

**Table A4:** Effects of an l-carnitine supplementation on calculated milk parameters of German Holstein cows challenged by an *intravenous* injection of lipopolysaccharides (LPS, 0.5 μg/kg BW) at the first day in week 1.

Week Relative to LPS Injection	Fat-Corrected Milk ^1^ [kg/d]	Energy-Corrected Milk ^2^ [kg/d]	Milk Energy ^3^ [MJ/kg]
**CON/CAR**			
**−1**	37.7/37.6	38.0/37.6	2.9/3.0
1	37.4/36.0	36.9/35.4	3.2/3.1
2	35.6/34.4	36.3/35.0	2.9/2.9
3	36.5/35.6	37.2/35.9	2.9/3.0
*p*-Value ^4^			
G	0.472	0.327	0.671
T	**0.003**	**0.002**	**<0.001**
G*T	0.750	0.848	0.153
**PSE** ^5^	0.756	0.676	0.046

Data are given as LS-Means for control group (CON) and l-carnitine group (CAR). ^1^ Fat-corrected milk = (milk fat [%] × 1.05 + 0.4) × milk yield [kg/d], ^2^ energy-corrected milk = milk yield [kg/d] × $\frac{1.05+0.38*\mathrm{milk}\;\mathrm{fat}\;\left[\%\right]+0.21*\mathrm{milk}\;\mathrm{protein}\;\left[\%\right]}{3.28}$, ^3^ milk energy = 0.38 × milk fat [%] + 0.21 × milk protein [%] + 0.95, ^4^ *p*-Value for group (G), time (T) and their interaction (G*T), ^5^ pooled standard error. Significant *p*-values are marked as bold.

**Table A5:** Effects of l-carnitine supplementation on concentration of triacyl-glycerides, total carbon dioxide (CO_2_), base excess in extracellular fluid compartment (Beecf), total calcium (Ca), ratio of total calcium and total phosphorus (Ca:P) and anion gap of German Holstein cows challenged by an *intravenous* injection of lipopolysaccharides (LPS, 0.5 μg/kg BW) at time point 0.

Day/Hours Relative to LPS Injection	Triacyl-Glycerides [mmol/L]	CO_2_ [mmol/L]	Beecf [mmol/L]	Ca [mmol/L]	Ca:P	Anion Gap ^1^ [mmol/L]
**CON/CAR**						
**−1**	0.13/0.130	32.72/33.04	7.07/7.48	2.30/2.35	1.58/1.71	8.30/9.75
0.5	0.17/0.18	32.93/30.16	7.27/4.63	2.19/2.15	1.52/1.67	8.17/10.67
1	0.09/0.10	29.44/29.76	3.26/3.55	2.04/1.96	1.67/1.69	9.37/10.75
2	0.09/0.10	29.00/30.12	2.76/4.02	1.91/1.70	1.76/1.61	8.14/8.08
3	0.08/0.08	29.31/30.23	3.71/4.84	1.60/1.54	1.99/1.92	7.77/9.00
4	0.08/0.08	24.88/26.37	−0.80/0.73	1.60/1.51	2.60/2.30	9.63/10.92
6	0.08/0.08	26.12/28.50	0.53/2.94	1.61/1.48	2.05/2.18	9.45/10.40
9	0.10/0.09	27.94/28.75	2.10/2.84	1.71/1.54	1.30/1.28	9.62/10.42
12	0.10/0.10	28.28/28.53	2.54/2.73	1.80/1.67	1.23/1.18	10.17/10.31
24	0.11/0.11	28.93/28.35	3.36/2.83	2.04/2.04	1.29/1.25	9.71/10.02
48	0.09/0.09	31.14/30.71	5.56/5.12	2.20/2.15	1.68/1.55	8.59/8.57
72	0.09/0.08	31.64/30.95	5.97/5.58	2.30/2.29	1.56/1.65	8.41/8.53
7	0.14/0.14	31.47/31.31	5.59/5.36	2.47/2.39	1.68/1.91	8.17/9.03
14	0.13/0.13	30.63/31.91	4.79/6.22	2.41/2.40	1.56/1.64	8.59/7.84
*p*-Value ^2^						
G	0.840	0.648	0.595	0.081	0.960	0.183
T	**<0.001**	**<0.001**	**<0.001**	**<0.001**	**<0.001**	**<0.001**
G*T	0.523	0.094	0.158	0.404	0.597	0.330
**PSE** ^3^	0.003	0.343	0.357	0.033	0.055	0.258

Data are given as LS-Means for control group (CON) and l-carnitine group (CAR). ^1^ Anion gap = [Na^+^] − ([HCO_3_^−^] + [Cl^−^]), ^2^ *p*-Value for group (G), time (T) and their interaction (G*T), ^3^ pooled standard error. Significant *p*-values are marked as bold.
